# Chronic psychosis associated with new hallucinogenic drug 25I-NBOMe

**DOI:** 10.1192/j.eurpsy.2023.1319

**Published:** 2023-07-19

**Authors:** O. Martin-Santiago, G. Guerra-Valera, C. Alario-Ruiz, N. Navarro-Barriga

**Affiliations:** Department of Psychiatry, Hospital Clinico Universitario de Valladolid, Valladolid, Spain

## Abstract

**Introduction:**

The presence of perceptual disturbances and psychotic symptoms associated with substance abuse are widely known. While the abuse of substances is becoming more widespread, there is a general perception that their use entails fewer risks. 25I-NBOMe is a recently introduced hallucinogenic drug producing visual hallucinations and euphoria. Although people consume it like LSD, its chemical structure is different to LSD. 25I-NBOMe is related to other phenylethylamine derivatives (amphetamines and mescaline).

**Objectives:**

Present a clinical case of psychosis triggered after the consumption of new emerging drugs and highlight that the extension of their consumption in the general population, especially in the most vulnerable, can trigger prolonged psychotic symptoms.

**Methods:**

We present a clinical case report of a subject who developed perceptual disturbances and paranoid symptoms. These lasted for months.

**Results:**

We describe the case of a 30-year-old man who required psychiatric admission after a single NBOMe intake five months earlier. He began with self-referential experiences and delusional ideas of prejudice, persecution and control in social networks. For months, intrusive images appeared in the form of flashbacks. He remains isolated, hardly sleeps and is easily irritated. He previously worked and had a well social network. Since adolescence, he had occasionally used alcohol, cannabis and cocaine. An uncle was diagnosed with schizophrenia. Treatment with long-term injectable aripiprazole started, reducing the symptoms and managing to recover work activity in a year.

**Conclusions:**

25I-NBOMe has its main activity as 5HT2 receptor agonism, which is associated with increased dopaminergic activity in the brain. Hallucinations, delusions, anxiety symptoms and depersonalization appear during acute consumption. However, some patients have developed a persistent hallucinatory chronic syndrome after consumption. As its use is expanding, it probably could increase the number of patients with induced chronic psychoses, especially those with greater susceptibility. One of the possible causes would be its analogous structure to other derivatives of phenylethylamine, which increase the risk of psychosis, and another would be the erroneous perception of being a less dangerous drug.

**Disclosure of Interest:**

None Declared

